# The neurocognitive mechanism linking temperature and humidity with miners’ working memory: an fNIRS study

**DOI:** 10.3389/fnhum.2024.1414679

**Published:** 2024-09-10

**Authors:** Chenning Tian, Hongxia Li, Shuicheng Tian, Fangyuan Tian, Hailan Yang

**Affiliations:** ^1^School of Safety Science and Engineering, Institute of Safety Management and Risk Control, Xi’an University of Science and Technology, Xi’an, China; ^2^School of Safety Science and Engineering, Institute of Safety and Emergency Management, Xi’an University of Science and Technology, Xi’an, China; ^3^School of Management, Xi’an University of Science and Technology, Xi’an, China

**Keywords:** cognitive performance, fNIRS, short-term visual memory task, temperature and humidity, working memory

## Abstract

**Background:**

In China’s coal mines, employees work in environments reaching depths of 650 m, with temperatures around 40°C and humidity levels as high as 90%, adversely affecting their health, safety capabilities, and cognitive functions, especially working memory. This study aims to explore different temperature and humidity conditions’ impact on neurocognitive mechanisms to enhance occupational health and safety.

**Methods:**

This study, conducted between June and August 2023, with 100 coalmine workers from the Hongliulin Mining Group, utilized functional near infrared spectroscopy (fNIRS) and short-term visual memory tasks to evaluate the effects of high temperatures and humidity on working memory by monitoring activity in the cerebral cortex. Behavioral data, and neurophysiological data were analyzed using Tukey’s HSD for significant differences and multiple regression to explore the impact of temperature and humidity. The β-values of Oxy-Hb for different regions of interest were calculated using General liner model (GLM), and the activation maps were plotted by NIRS_KIT.

**Results:**

High temperature and humidity (Condition IV) significantly depressed reaction times and working memory compared to other conditions, with temperature having a more pronounced impact than humidity on these cognitive measures (*p* < 0.05). Oxy-Hb concentration increased notably under Condition IV, emphasizing temperature’s influence on brain oxygen levels. ROI analysis revealed varied brain activation patterns. The activation of ROI A and B (prefrontal cortex) increased with the increase of temperature and humidity, while ROI C (supplementary motor area) was less sensitive to temperature, indicating the complex influence of environmental factors on brain function.

**Conclusion:**

This study highlights the important effects of temperature and humidity on cognitive performance and brain function, highlighting the need to optimize the environment of miners’ sites to improve productivity and safety.

## 1 Introduction

Employees in coal mines often need to work in humid and hot environment on daily job basis. In the present, the average mining depth in the China coal mine has reached 650 meters and even more, where the temperature at the mining face is around 40°C and the average humidity is as high as 90%. This sort of humid and hot environment resulted in worse worker’s physical health, decreased capabilities of safety operation, as well as cognitive performance. Among various cognitive functions, working memory plays a key role in efficient task performance in such environments. This short-term memory, is a cornerstone for performing complex cognitive operations and decision making (Baddeley, 1992; Zuri et al., 2013; Hemmatjo et al., 2017; Zhu et al., 2022). When miners need to react to new situations, fatigue caused by heat exposure can interfere with their ability to react quickly. Because the mining industry relies on heavy mobile equipment and other dangerous activities, such as detonating explosives, miners’ heat, sleepiness, and cognitive impairment are potential dangers to themselves and others (Legault, 2011). Understanding the neurocognitive mechanisms of how hot and humid conditions affect working memory helps improve working conditions for miners, which is a key element to improving occupational health and safety standards in mining operations.

Compared to neurophysiological parameters, cognitive function is more acutely responsive to humid and hot environments (Gaoua, 2010). The increase in core temperature due to heat stress is typically accompanied by a rise in brain temperature, which can impair neural activity and potentially lead to brain dysfunction (Kiyatkin, 2005). Miners working in complex underground environments often experience significant memory impairment, displaying cognitive characteristics similar to those observed in patients with mild cognitive impairment (Çelik et al., 2024). High fever also adversely affects both working memory and short-term memory, primarily through abnormal frontal lobe activity (Racinais et al., 2008). But many studies have not particularly paid attention to hot and humid conditions as separate factors but rather included them in the studies on extreme environmental conditions or discussed high temperatures alone, which may mix up the comprehension of the exact implications that heat and humidity would have on working memory.

Traditional researchers have relied on self-report methods and performance on laboratory tasks to understand and predict human behavior. However, these indicators offer limited predictive power regarding behavior in specific contexts. In contrast, neuroimaging serves as a complementary approach, uncovering connections between neural activity and medium- to long-term, ecologically valid outcomes within a laboratory setting (Berkman and Falk, 2013; Herold et al., 2018). Some researchers have made brain function studies through neural imaging, which is done by means of functional magnetic resonance imaging (fMRI). These experiments usually have very strict limitations on the possible activities of the participants and on the conditions in which they are performed, which makes it very difficult to simulate the environment of real-life mining (Logothetis, 2008). It has been demonstrated that using functional near-infrared spectroscopy (fNIRS) to measure miners’ alertness in simulated mine heat and humidity environments is feasible (Tian et al., 2024). To further investigate the effects of heat and humidity on miners, we combined fNIRS as a neuroimaging tool with short-term visual memory tasks. This study aims to explore how high temperature and high humidity in coal mines affect working memory-related neurocognitive mechanisms by monitoring cerebral cortex activity. Compared with other tools, fNIRS is not intrusive, can measure actual brain activity in free-world environment, and its high temporal resolution makes it an appropriate tool for research of cognition processes in natural surroundings (Providência and Margolis, 2022). fNIRS has been widely used to locate brain activity during tasks (Taga et al., 2003; Nakano et al., 2009). In the field of safety research, scholars from various domains, including driving (Liu et al., 2016), construction (Hu et al., 2018), and aviation (Dehais et al., 2018) have extensively employed fNIRS to investigate workers’ unsafe behaviors.

In order to comprehensively understand the neurocognitive mechanisms underlying miners’ unsafe behaviors, it is imperative to focus on the relationship between temperature, humidity, and miners’ working memory. This study utilizes fNIRS to measure the cerebral blood oxygen level and the behavioral reactions of miners performing short-term working memory tasks at hot and humid workstations. The intent is to understand the effects of hot and humid working environment on brain activation and physiological processes in miners. Which helps formulate intervention strategies and techniques aimed at containing the risks and improving the mental state of miners.

## 2 Materials and methods

### 2.1 Participants

This study included 100 coalmine workers (age 34 ± 6) from the Hongliulin Mining Group who were randomly chosen and participated in the experiment between June and August 2023. The miners were composited into 4 experimental groups randomly, each in groups of 25 people.

All participants were right-handed and had no addiction to smoking or drinking alcohol. They were all with normal or corrected vision, with their eyes unaffected by any color blindness or other eye diseases. Moreover, all participants ensured they got adequate sleep and refrained from taking psychotropic medication before the experiment. Additionally, all participants took part in the experiment in the morning to minimize the impact of circadian rhythms on working memory.

The participants were briefed that the study evaluates the effect of a certain approach on cognitive processes. They were not, though, informed about the design or the specific composition of the groups. Before the experiment, each participant was clearly informed that they had the right to drop out or to stop the experiment, and nobody quit. Before the research, each participant signed a written informed consent. This study received ethical approval from the Ethics Committee of Xi’an University of Science and Technology (Approval number: XUSTETHP002023052302) and follows the latest version of the Declaration of Helsinki.

### 2.2 Experiment condition

**Figure 1:**
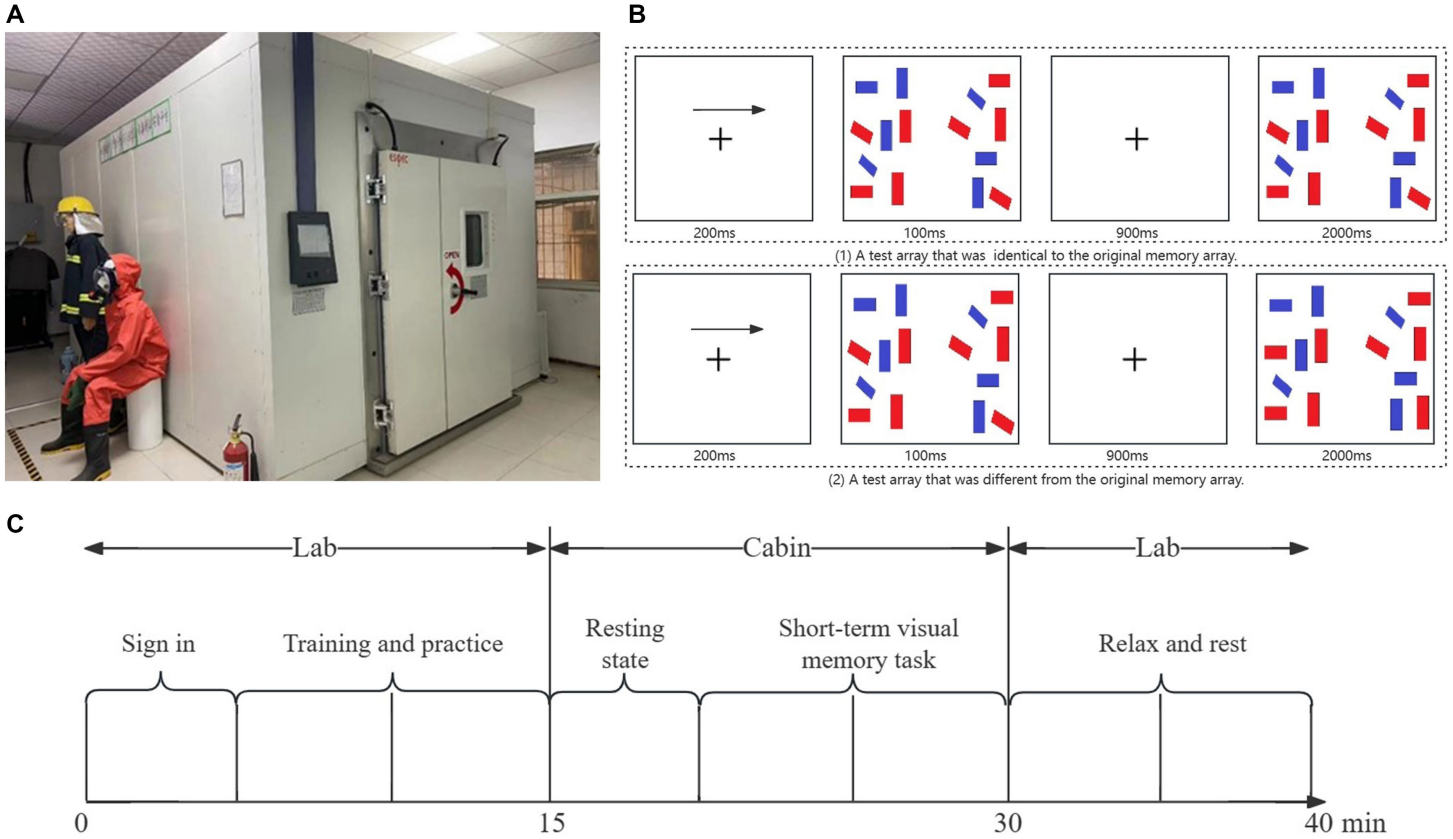
**(A)** The experiment cabin. **(B)** An illustration of the experimental task. **(C)** An illustration of experimental procedure.

The experimental location was chosen in Safety and Emergency Management Laboratory of Xi’an University of Science and Technology. A walk-in temperature and humidity cabin (SEWTH-A-290H) was built in the laboratory (Figure 1A). The interior dimension of the laboratory is 3* 2.4* 4 m, and the external dimension is 3.2*2.7*5.6 m. Temperature setting range − 20°C ~ +90°C, humidity control range 20% ~ 95% R.H. The cabin was a dark environment, and the only light source was the screen light of the experimental machine. The screen light source was measured by a hand-held illuminator (SuWei SW6023), and the screen light brightness was 60 lux. The subjects sat in front of the only table (1,000 × 500 × 700 mm) in the experimental cabin and performed the experimental tasks through the experimental machine on the table (screen size 19 inches; Screen ratio 16:10; Resolution 1,680 × 1,050). The average cabin noise volume is 71 decibels.

In the revised 2016 edition of the “Coal Mine Safety Regulations of China,” it is stated that when the air temperature on the mining face exceeds 26°C and the temperature in the equipment chamber exceeds 30°C, the working hours of personnel at overheated locations must be shortened, and thermal stress management measures must be taken. Compared to the maximum allowable temperatures in coal mines in other countries around the world, the environmental evaluation criteria in China’s coal mines are stricter. Statistics on underground working place humidity in China (Peng, 2018) showed that the average relative humidity of coal mine working faces is 89%.

According to the actual temperature and humidity conditions in Chinese coal mines and the maximum working environment parameters supported by experimental measurement equipment, the specific experimental environment settings are presented in Table 1.

**Table 1:** Experimental conditions and number of participants.

Conditions	Air temperature	Relative humidity	Number of participants^1^	Mean age
Condition I	25°C (77°F)	55%	24/25	32.52 (±4.38)
Condition II	35°C (95°F)	55%	20/25	32.32 (±4.86)
Condition III	25°C (77°F)	85%	20/25	33.16 (±5.33)
Condition IV	35°C (95°F)	85%	25/25	32.68 (±4.48)

1 “Number of Participants” refers to the number of valid participants/actual participants.

### 2.3 Short-term visual memory task

Short-term visual memory task is a special visual memory task used to study working memory. Over the past 5 years, researchers have used short-term visual memory tasks to study working memory in terms of measuring model (Oberauer, 2023), sensitivity to precise task (Ades and Mishra, 2022), changes in brain activity during memory task (Sasabe and Hagiwara, 2018), and reliability and stability of working memory (Xu et al., 2018). Working memory is essential for everyday human behaviors such as language understanding, learning, and reasoning, and is known as the “working space” of consciousness (Baddeley, 2003). Although the long-term memory of humans has almost infinite capacity, working memory has a very limited capacity (Luck and Vogel, 2013), especially visual working memory, which usually can only hold 3 or 4 objects or chunks (Carlisle et al., 2011).

Pashler first established a calculation model based on hit rate (h) and false alarm rate (f), and obtained the upper limit of the average number of items held in the memory of subjects (the capacity of working memory), represented as K = N * (h − f)/(1 − f) (Pashler, 1988), where N is the number of items presented. When N is less than the capacity K, all items can be remembered; when N exceeds K, the value of K can be deduced using this formula. Later, Cowan pointed out that Pashler’s formula only applies when all the probe stimuli are present at the locations of all memory items, and if only one probe stimulus is present (the location matches one item in the memory sequence), the formula should be revised to K = N *(h − f) (Cowan, 2001). We use a formula developed by Pashler and improved by Cowan to calculate visual memory capacity.

Given the importance of working memory in the field of neuropsychology and the aim of exploring its neurophysiological relevance with more accessible and adaptable tools, this study first replicated Vogel’s experiment I (Vogel et al., 2005; Figure 1B) using near-infrared spectroscopy (fNIRS). On the basis of the experiment, we investigated the working memory performance of the miners under different humidity and temperature conditions. Since attention and working memory have always been 2 interrelated cognitive functions (Ku, 2018), we recorded the reaction time and working memory capacity of the miners, respectively.

### 2.4 Experimental procedures

The short-term visual memory task utilized in this study was programmed using E-Prime 3.0 software. The experiment procedure is detailed as Figure 1C: Participants arrive at the lab and first complete the sign-in procedure. The researcher then explained the experimental procedure in detail. Participants were told that they would perform the task under specific temperature and humidity conditions, but were not told about specific environmental parameters and received brief task training and practice. The participants then entered the cabin, sat in a comfortable position, and were assisted by the researcher in wearing a portable fNIRS device. After 5 min of resting, the participants began a 10-min short-term visual memory task that included approximately 180 judgements. The data recorded by the FNIRS device in this case is for 15 min, with 5 min of rest and 10 min spent on the task. After completing the task, the participants were guided to rest in a normal temperature and humidity environment for energy recovery and temperature and humidity regulation.

### 2.5 Data acquisition

fNIRS recordings were conducted under two conditions: (1) during a resting state and (2) during the short-term visual memory task. In the resting state, participants sat quietly in a dimly lit room with their eyes closed but remained awake for 5 min, during which baseline physiological measurements were recorded. During the short-term visual memory task, fNIRS recordings were taken continuously throughout the task, encompassing both task execution periods and rest intervals.

The experiment utilized the Cortivision Photon Cap (model C20), a portable near-infrared optical brain imaging system, along with the Cortivision Pathfinder to measure hemodynamic data in the cerebral cortex. The Cortivision Photon Cap employed an advanced version of the “10–5 system” (Oostenveld and Praamstra, 2001) for electrode placement, surpassing the traditional “10–20 system” and “10–10 system” used in EEG.

**Figure 2:**
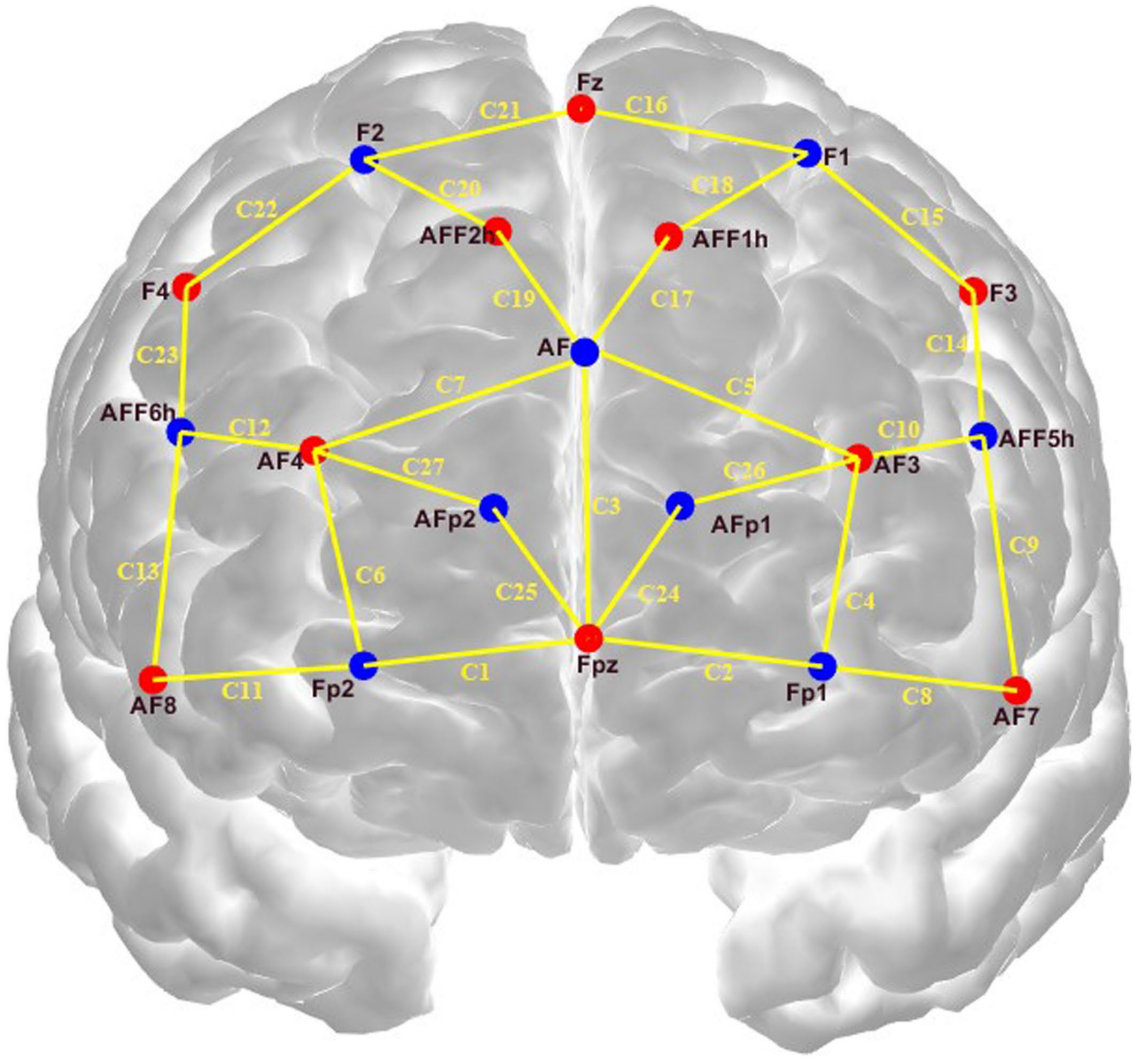
Distribution of optical electrodes.

This experiment covered a total of 27 channels near the prefrontal cortex, consisting of 10 light sources and 9 detectors. The spacing between the light sources and detectors for long-distance channels was maintained at approximately 30 millimeters, while for short-distance channels it was approximately 20 millimeters. A custom-designed holder ensured these distances remained constant. The sampling rate was 5–6 Hz. Figure 2 illustrates the electrode positions: light sources are red, detectors are blue, and fNIRS channels are indicated by yellow lines. Additionally, Table 2 provides details on the Brodmann areas, corresponding anatomical locations, and the defined regions of interest (ROIs) used in this study. ROI A and B are the prefrontal lobes (PFCS), specifically the dorsolateral prefrontal lobe (DLPFC), which is considered a cognitive region and is primarily involved in cognitive behavior (such as decision-making). ROI C is the premotor cortex and the auxiliary motor cortex, which are mainly involved in initiating and executing movement (Pooladvand and Hasanzadeh, 2022).

**Table 2:** Channels Brodmann areas, associated anatomic location, and ROI labels.

Channels	Brodmann areas	Anatomic location	ROIs
1,3,6,7,11,12,13,19,20,21,22,23,25,27	8, 9, 10	Prefrontal cortex (right)	A
2,3,4,5,8,9,10,14,15,16,17,18,24,26	8, 9, 10	Prefrontal cortex (left)	B
5,7,16,17,18,19,20,21,26,27	6	Supplementary motor cortex	C

### 2.6 Data analysis

After ruling out abnormal data due to equipment failure or excessive head movement, we obtained near-infrared spectral data for 89 miners. During data processing, we excluded participant data with more than 20% error signals.

We took the time required to react (RT) from the participants, and then we calculated working memory capacity (K). To investigate the effect of temperature and humidity on working memory, Tukey’s HSD was used to compare the reaction time and working memory capacity of the subjects under 4 conditions, respectively. To further confirm that temperature has a greater effect on RT and K of miners than humidity, we used a multiple linear regression model to analyze RT and K under 4 conditions, respectively. The multiple linear regression model was established by MATLAB. Temperature and humidity are extracted as independent variables, RT and K as dependent variables.

Data were preprocessed using the NIRS_KIT software (version 3.0_Beta) (Hou et al., 2021). The data type was designated as type13 and the PPF value was set to [6 6]. The modified Beer–Lambert law was applied to convert raw light intensity data into changes in oxygenated hemoglobin (Oxy-Hb) and deoxygenated hemoglobin (Deoxy-Hb) concentrations (Cope and Delpy, 1988). Detrending was done in the first step, a polynomial regression model is used to estimate a linear or non-linear trend and then subtracts it from the raw hemoglobin concentration signal. In the second step, the time derivative distributed repair (TDDR) method was employed for motion correction (Fishburn et al., 2019). A fast Fourier transform-based filter (0.01–0.1 Hz) was applied to transform the time series of each channel into the frequency domain, filter the signal, and then transform it back into the time domain. Finally, shallow noise was recorded using short-range reference channels and removed from the neural record using regression.

After preprocessing, individual-level statistical analyses were performed using a general linear model (GLM) to examine task-related neural activation. The GLM enabled the calculation of the hemodynamic response function (HRF) from a linear combination of different components, providing an accurate characterization of brain activation responses (Yücel et al., 2021). The steps included: (1) Signal Selection: Changes in Oxy-Hb concentration were used as the primary indicator of brain activity due to their lower susceptibility to confusion compared to Deoxy-Hb (Strangman et al., 2003). The default Canonical hemodynamic response function (HRF) was used; (2) Design Matrix: The matrix was designed according to each subject’s stimulus presentation and reaction times, with the “different” option selected in Design Inf type. Each row represented a time point, and each column represented an effect or explanatory variable; (3) Contrast Vector: A contrast vector ([1–1]) was used to estimate the mean signal value under multiple conditions or the amplitude difference between two conditions; (4) Short-distance Channels: These were added to the GLM as covariates. Changes in Oxy-Hb concentration levels for each channel were simulated by convolving a typical HRF with a binary time series containing stimulus timing.

To compare changes in Oxy-Hb concentrations across various environments, data from the “Oxydata” folder were organized and pooled by condition. The average Oxy-Hb concentration was calculated for each channel, excluding six short-distance channels. These averaged concentrations were subjected to Tukey’s HSD test at a 0.05 significance level to evaluate differences under different conditions. Multiple linear regression models were employed to investigate the effects of temperature and humidity on Oxy-Hb concentrations among miners. Additionally, ANCOVA was performed to determine the independent effects of age on Oxy-Hb concentrations, apart from temperature and humidity.

To observe differences in response to stimuli under different environmental conditions across three ROIs, “Oxy\con1” data after GLM calculations were used. Beta regression coefficients were obtained as indicators of brain activity for each channel, averaged according to channel classification, and visualized. A Wilcoxon signed-rank test was used to examine differences between the two ROIs in different conditions.

## 3 Results

We established three ANCOVA models to analyze the relationship between reaction time, error rate, Oxy-Hb concentrations and age respectively, so that the influence of age on these three variables can be effectively controlled. The ANCOVA analysis results are shown in Table 3.

**Table 3:** ANCOVA analysis results of the effects of age on RT, ER, and Oxy-Hb.

	Reaction time				Error rate			
	Estimate	SE	*T*	*P*-value	Estimate	SE	*T*	*P*-value
(Intercept)	382.52	5.390	71.02	0	11.571	5.664	2.04	0.0527
Age	−0.2037	0.164	−1.241	0.227	−0.0788	0.172	−0.456	0.652
R-squared	0.0628				0.00898			
Adjusted R-squared	0.022				−0.0341			

	Oxy-Hb concentrations(e-08)			
	Estimate	SE	*T*	*P*-value
(Intercept)	4.1988	8.1704	−0.514	0.6122
Age	−3.2588	0.2491	1.3083	0.2037
R-squared	0.0693			
Adjusted R-squared	0.0288			

SE stands for Standard Error.

The results above indicate that age has no significant impact on RT or ER or Oxy-Hb concentrations in the current data. Therefore, in subsequent analyses and reporting for this study, the influence of other factors on RT and ER and Oxy-Hb concentrations can be considered, instead of the influence of age.

We observed significant differences in both behavioral and physiological measures between conditions. Table 4 presents the raw means, standard deviations of response times, working memory capacity (K) and Oxy-Hb concentration in four conditions.

**Table 4:** Mean value and standard deviation of response times, working memory capacity (K) and Oxy-Hb concentration.

Condition	Response times		Working memory capacity (K)		Oxy-Hb concentration(E-08)	
	M	SD	M	SD	M	SD
I	472.99	118.13	3.91	0.33	1.16	3.25
II	559.96	125.35	3.48	0.59	3.59	3.51
III	509.96	284.01	3.72	0.41	3.19	2.99
IV	638.95	206.16	3.13	0.48	7.04	4.84

### 3.1 Behavioral results

The response times and working memory capacity (K) under the 4 conditions were subjected to Tukey’s HSD test, the results are displayed in Table 5.

**Table 5:** Tukey’s HSD test results for reaction time and working memory capacity (K).

Cond	Reaction time				Working memory capacity (K)			
	MD	Lower CI	Upper CI	*p*-value	MD	Lower CI	Upper CI	*p*-value
I vs. II	−107.8525	−86.1002	−64.3479	<0.05	−0.0448	−0.0792	−0.0104	<0.05
I vs. III	−70.9411	−49.1889	−27.4366	<0.05	−0.0208	−0.0552	0.0136	0.3941
I vs. IV	−197.3054	−175.5531	−153.8009	<0.05	−0.0804	−0.1148	−0.0460	<0.05
II vs. III	15.1591	36.9113	58.6636	<0.05	0.0240	−0.0104	0.0584	0.2683
II vs. IV	−111.2052	−89.4529	−67.7007	<0.05	−0.0356	−0.0700	−0.0012	<0.05
III vs. IV	−148.1165	−126.3643	−104.6120	<0.05	−0.0596	−0.0940	−0.0252	<0.05

The analysis indicated that, compared to Conditions II, III, and IV, the response time in Condition I was significantly reduced. The mean differences were − 107.8525, −70.9411, and − 197.3054, respectively, with *p*-values all less than 0.05, signifying that reaction time in Condition I was faster with statistical significance. This suggests that elevated levels of temperature and humidity may impede cognitive reactions. Regarding working memory capacity, Condition I once again demonstrated a statistically significant higher capacity compared to Condition II (*p* = 0.0053) and Condition IV (*p* < 0.001). The comparison between the first and third groups was not statistically significant (*p* = 0.3941), nor was the comparison between the second and third groups (*p* = 0.2683). The results indicate that the most extreme conditions (IV) exhibit the most pronounced association with a reduction in working memory capacity.

In summary, Condition I provides the most advantageous environment for maintaining faster reaction times and higher working memory capacities. Condition IV exhibits the poorest performance in these two cognitive indicators. This may be attributed to the increased consumption of cognitive resources and the response to environmental stress. The independent effects of temperature and humidity are relatively minor, yet they exhibit a notable combined effect.

To further substantiate the hypothesis that temperature has a more significant impact than humidity on miners’ reaction times and error rates, a multivariate linear regression model should be utilized to analyze the reaction times of the 4 groups of miners. The multivariate linear regression model is constructed using MATLAB, with the results presented in Table 6.

**Table 6:** Regression results for reaction time and error rate.

	Reaction time				Working memory capacity (K)			
	Estimate	SE	t-Stat	*p*-value	Estimate	SE	t-Stat	*p*-value
Intercept	70.262	24.646	2.851	0.00532	5.745	0.357	16.113	3.55e-29
Temperature	10.623	0.642	16.523	5.91e-30	−0.051	0.009	−5.520	2.82e-07
Humidity	2.310	0.214	10.782	2.77e-18	−0.009	0.003	−2.961	0.003
R-squared	0.801				0.688			
Adjusted R-squared	0.796				0.673			
F-Statistic	195				19.6			
*p*-Value	1.11e-34				6.99e-08			

The result reveals that both temperature and humidity have a statistically significant impact on reaction times and working memory capacity. Temperature has a considerable positive effect on response time, with an estimated increase of 10.623 units for each additional degree Celsius (*p* < 0.0001). Humidity also has a positive effect on response time, with each additional percentage point of humidity increasing response time by 2.3107 units (*p* < 0.0001). The coefficient for temperature is associated with a slight decline in working memory capacity, with a coefficient of −0.05134 per degree Celsius (*p* < 0.0001). Similarly, humidity has a negative effect, with each additional percentage point decreasing working memory capacity by −0.00918 units (*p* = 0.0038538).

### 3.2 fNIRS results

As shown in Table 7, no significant differences were observed when comparing Condition I with Condition II and Condition III (*p >* 0.05). However, a significant reduction in Oxy-Hb concentration was noted when Condition I was compared to Condition IV (*p* < 0.05). Furthermore, Condition II compared to Condition III showed no significant difference (*p* > 0.05), whereas a comparison between Condition II and Condition IV indicated a significant decrease in Oxy-Hb concentration for Condition IV (*p* < 0.05). Similarly, Condition III compared to Condition IV also resulted in a significant decrease (*p* < 0.05).

**Table 7:** Results of Tukey’s HSD test for Oxy-Hb concentration.

Cond	MD	Lower CI	Upper CI	*p*-value
1vs2	−2.4345E-08	−5.0764E-08	2.0737E-09	>0.05
1vs3	−2.0275E-08	−4.6693E-08	6.1442E-09	>0.05
1vs4	−5.8832E-08	−8.5251E-08	−3.2413E-08	<0.05
2vs3	4.0705E-09	−2.2348E-08	3.0489E-08	>0.05
2vs4	−3.4487E-08	−6.0906E-08	−8.0684E-09	<0.05
3vs4	−3.8558E-08	−6.4976E-08	−1.2139E-08	<0.05

To test the hypothesis that the influence of temperature on the cerebral blood oxygen concentration of miners is greater than the influence of humidity on the same. A multiple-regression model was developed (Table 8).

**Table 8:** Regression results for Oxy-Hb concentration.

	Estimate	SE	t-Stat	*p*-value
Intercept (*E-08)	−12.078	2.7423	−4.4042	2.5649e-05
Temperature (*E-08)	0.3145	0.0715	4.3957	2.6512e-05
Humidity (*E-08)	0.0913	0.0238	3.8272	0.00022053
R-squared	0.744			
Adjusted R-squared	0.73			
F-Statistic	17			
*p*-Value	4.07e-07			

The temperature coefficient is 0.315 (*p* < 0.05), indicating a positive correlation between temperature and oxygen concentration. Specifically, for each unit increase in temperature, the oxygen concentration is anticipated to rise by 0.3145 units. The humidity coefficient is 0.0913 (*p* < 0.05), suggesting a positive correlation with oxygen concentration as well. For each unit increase in humidity, oxygen concentration is expected to increase by 0.0913 units. Which means the influence of temperature on Oxy-Hb concentration is greater than that of humidity.

**Figure 3:**
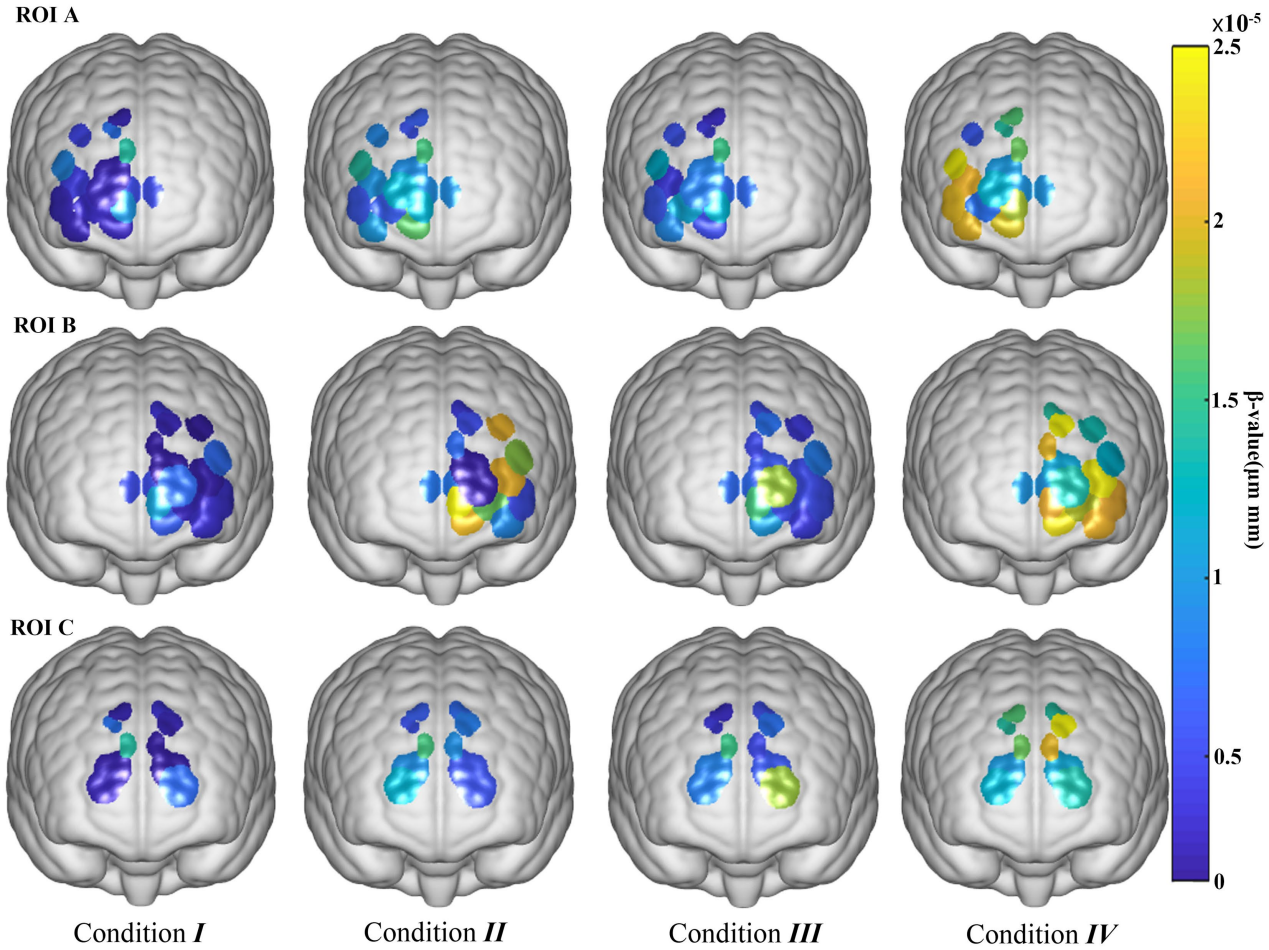
Visualization of the activation of ROIs under 4 conditions.

To find out the activation of ROI in 4 Conditions, we performed Wilcoxon signed rank test on the β values of Oxy-Hb of the 3 ROIs and made visualization diagrams (Table 9; Figure 3). To avoid the “multiple test problem” that occurs when multiple comparisons are made, that is, the probability of false positives (false positives) increases, we used the Bonferroni correction method to adjust the Wilcoxon test results for multiple comparisons. Comparing *p*-values and corrected *p*-values, it can be found that the correction process significantly raises the significance threshold and reduces the possibility of false positive results.

**Table 9:** Wilcoxon signed-rank tests results for three ROIs.

ROI	Cond	Mean (e-06)	Std (e-06)	Z			*p*-value			Corrected *p*-value		
				II	III	IV	II	III	IV	II	III	IV
A	I	5.23	3.57	−1.10	2.19	−1.83	0.0004	0.0067	0.0001	0.0022	0.0403	0.0007
	II	1.02	3.49		32.13	0.37		0.0083	0.0034		0.0498	0.0205
	III	7.94	3.6			0.00			0.0012			0.0073
	IV	16.1	6.86									
B	I	4.22	2.76	−1.46	−1.83	−1.83	0.0002	0.0001	0.0001	0.0015	0.0007	0.0007
	II	12.1	5.2		31.40	1.46		0.0134	0.0040		0.0806	0.0242
	III	7.66	4.22			−0.73			0.0006			0.0037
	IV	18.2	6.04									
C	I	4.47	4	−0.37	0.00	−1.83	0.0137	0.0195	0.0020	0.0820	0.1172	0.0117
	II	8.46	3.24		13.51	−1.83		0.1602	0.0039		0.9609	0.0234
	III	8.09	4.8			−0.37			0.0137			0.0820
	IV	15.9	6.01									

Yellow in the figure indicates higher activation levels, while blue indicates lower activation levels. From the 3D plot, humidity does not appear to have as significant an effect on activation as temperature, as can be seen from the corrected *p*-values in Table 9. Specifically, in ROI A, the *p*-values for all comparisons were less than 0.05, indicating a significant change. However, the *p*-value of condition III compared with condition 1 and condition II is significantly higher than the other conditions. This is also evident from Figure 3, where the degree of color change in Condition III is less pronounced than in condition I and Condition II. Bonferroni correction results showed that the difference between ROI B and ROI A was that the corrected *p*-value between conditions II and III was greater than 0.05, and the corrected *p*-value between other conditions was less than 0.05. In addition, the overall activation level of ROI B was higher than that of ROI A. In ROI C, only condition I and condition IV, and condition II and condition IV have corrected *p*-values less than 0.05. As can be seen from the figure, the activation degree is less than ROI A and ROI B.

## 4 Discussion

In this study, we explored the performance of behavioral indicators (reaction time, working memory capacity) and physiological indicators (Oxy-Hb concentration, ROI activation) of short-term visual memory tasks in miners under different temperature and humidity conditions. To our knowledge, this is the first time to study the effects of specific temperature and humidity conditions in coal mines on the neurocognitive function by using fNIRS, especially working memory, of coal miners from the perspectives of neuroscience and behavioral science.

Initially, we conducted an analysis of behavioral data, specifically focusing on the reaction time and working memory capacity in the short-term visual memory task. The findings show that there are significant changes in the reaction time and working memory capacity of miners under 4 conditions, especially under Condition IV (high temperature and high humidity), this effect is most obvious, manifested by a significant decline in working memory capacity and an increase in reaction time. This suggests that increases in temperature and humidity may be detrimental to miners’ cognitive performance, particularly affecting judgment and performance of working memory tasks. This finding is similar to the research reported by Zhao et al. (2009). In addition, we found that temperature has a more significant impact on reaction time and error rate than humidity, and further confirms the combined effects of temperature and humidity on cognitive function, which is consistent with the research results of Shi et al. (2013).

In the second part of the study, we focused on sustained brain activity by analyzing fNIRS data, specifically the activation levels of Oxy-Hb concentration and ROI. When the Oxy-Hb concentration was compared in pairs under different conditions, it was found that the Oxy-Hb concentration under high temperature and high humidity conditions was significantly different from the other 3 conditions, while the difference between the latter conditions was not statistically significant. The results of multiple linear regression analysis highlighted the significant influence of temperature and humidity on Oxy-Hb concentration, and the significant positive correlation between temperature and Oxy-Hb concentration may reflect the increase of metabolic activity at high temperature, requiring more oxygen supply. The positive correlation of humidity suggests that changes in atmospheric conditions can affect oxygen concentrations, although the mechanisms behind this may require further study. This suggests that high temperatures and high humidity may lead to cognitive decline, consistent with the findings of Zhang et al. (2017), and further confirms the combined effects of temperature and humidity (Shi et al., 2013).

To find the activation of ROI, visualizations are used to describe the activation levels of the channels corresponding to the three ROIs. We observed the highest level of channel activation under condition IV of all ROIs. In all ROIs, temperature and humidity showed significant effects on channel activation, with temperature having a more significant effect.

Regions of Interest (ROI) A and B are part of the prefrontal cortex (PFC), contributing to the frontal attention network, which is responsible for the allocation and maintenance of attentional resources (Nogueira et al., 2022). The PFC is considered a pivotal area in the brain, providing biased signals to other regions to activate neural pathways and establish the input–output patterns necessary for specific tasks (Miller and Cohen, 2001). Consequently, changes in PFC activation are related to cognitive processes, information processing, and decision-making during task execution. In this experiment, increased temperature and humidity lead to higher cognitive load. This occurs because individuals maintain better attention and process environmental information more efficiently in relatively comfortable conditions (Condition I), resulting in lower brain activation. Increased cognitive load can be interpreted as higher task difficulty. The study uses the same experimental paradigm, with participants performing identical tasks under varying environmental conditions. When temperature and humidity are high, participants exert more effort to complete the same task, indicating increased workload or work intensity. This places additional demands on the limited global workspace, reducing available resources for cognitive tasks. According to the global workspace theory, when overall attentional resources are insufficient, cognitive task performance deteriorates (Fairclough et al., 2018). In such situations, decreased attention or physical fatigue increases the likelihood of task failures or omissions, potentially leading to accidents in practical and everyday scenarios.

Compared to ROI A and B, the activation of channels in ROI C is less pronounced in response to humidity changes and shows relatively less sensitivity to temperature variations. ROI C primarily includes the supplementary motor area (SMA), which is crucial for decision processes in motor initiation and action execution. Results indicate a rising trend in blood oxygenation levels in ROI C from Condition I to Condition IV. Previous research has shown that task intensity directly regulates cerebral hemodynamics, with higher task intensity resulting in more significant brain activation (Rasmussen et al., 2007). The SMA is mainly involved in planning and coordinating movements, particularly in generating complex movement sequences and spontaneous actions. In contrast, the prefrontal cortex (especially the right PFC) is more extensively involved in higher-order cognitive functions such as decision-making, planning, inhibitory control, and working memory. Considering that the short-term visual memory task does not involve complex motor planning and coordination, this explains why SMA activation is less pronounced than PFC activation.

## 5 Conclusion

This comprehensive study underscores the critical need for optimizing environmental conditions to enhance cognitive performance and brain function. Our findings indicate that reduced reaction times and improved working memory capacities are significantly associated with cooler, less humid conditions (Condition I), highlighting the detrimental impact of high temperature and humidity on these cognitive functions. Moreover, the differential activation patterns observed across various brain regions further elucidate the complex interplay between environmental factors and cerebral activity, with the most extreme conditions (Condition IV) markedly affecting cerebral functioning. This research suggests that temperature holds a more substantial influence over cognitive performance than humidity, a crucial consideration for designing workplaces or environments where cognitive demands are high. The significance of these findings extends beyond the immediate study context, offering valuable insights for occupational health, workplace safety, and the broader field of environmental psychology.

## Data Availability

The raw data supporting the conclusions of this article will be made available by the authors, without undue reservation.
