# Corrigendum: The neurocognitive mechanism linking temperature and humidity with miners' working memory: an fNIRS study

**DOI:** 10.3389/fnhum.2024.1505772

**Published:** 2024-10-16

**Authors:** Chenning Tian, Hongxia Li, Shuicheng Tian, Fangyuan Tian, Hailan Yang

**Affiliations:** ^1^School of Safety Science and Engineering, Institute of Safety Management and Risk Control, Xi'an University of Science and Technology, Xi'an, China; ^2^School of Safety Science and Engineering, Institute of Safety and Emergency Management, Xi'an University of Science and Technology, Xi'an, China; ^3^School of Management, Xi'an University of Science and Technology, Xi'an, China

**Keywords:** cognitive performance, fNIRS, short-term visual memory task, temperature and humidity, working memory

In the published article, the reference for Rasmussen et al., 2007 was incorrectly written as “Rasmussen, P., Dawson, E. A., and Nybo, L. (2007). Capillary-oxygenation-level-dependent near-infrared spectrometry in frontal lobe of humans[J]. *J. Cereb. Blood Flow Metab*. 27, 1082–1093.” It should be “Rasmussen, P., Dawson, E. A., Nybo, L., Van Lieshout, J. J., Secher, N. H., and Gjedde, A. (2007). Capillary-oxygenation-level-dependent near-infrared spectrometry in frontal lobe of humans. *J. Cereb. Blood Flow Metab*. 27, 1082–1093.”

The authors apologize for this error and state that this does not change the scientific conclusions of the article in any way. The original article has been updated.

